# Fiber Bragg Gratings, IT Techniques and Strain Gauge Validation for Strain Calculation on Aged Metal Specimens

**DOI:** 10.3390/s110101088

**Published:** 2011-01-19

**Authors:** Ander Montero, Idurre Saez de Ocariz, Ion Lopez, Pablo Venegas, Javier Gomez, Joseba Zubia

**Affiliations:** 1 Aeronautical Technologies Center, Technologic Park of Alava., C/Juan de la Cierva 1., Miñano (Alava) 01510, Spain; E-Mails: iocariz@ctaero.com (I.S.O.); ilopez@ctaero.com (I.L.); pvenegas@ctaero.com (P.V.); 2 Department of Electronics and Telecommunications, Faculty of Engineering, University of the Basque Country, Alda Urquijo, s/n. 48013 Bilbao, Spain; E-Mail: joseba.zubia@ehu.es (J.Z.)

**Keywords:** Structural Health Monitoring (SHM), Non Destructive Evaluation (NDE), Fiber Bragg Grating (FBG), Infrared Thermography (IT), strain, Relative Humidity (RH), temperature, aging

## Abstract

This paper studies the feasibility of calculating strains in aged F114 steel specimens with Fiber Bragg Grating (FBG) sensors and infrared thermography (IT) techniques. Two specimens have been conditioned under extreme temperature and relative humidity conditions making comparative tests of stress before and after aging using different adhesives. Moreover, a comparison has been made with IT techniques and conventional methods for calculating stresses in F114 steel. Implementation of Structural Health Monitoring techniques on real aircraft during their life cycle requires a study of the behaviour of FBG sensors and their wiring under real conditions, before using them for a long time. To simulate aging, specimens were stored in a climate chamber at 70 °C and 90% RH for 60 days. This study is framed within the Structural Health Monitoring (SHM) and Non Destructuve Evaluation (NDE) research lines, integrated into the avionics area maintained by the Aeronautical Technologies Centre (CTA) and the University of the Basque Country (UPV/EHU).

## 1. Introduction

The latest trends in aircraft design are aimed at decreasing the costs associated with aircraft maintenance and operation, while ensuring their maximum safety. For this reason SHM research with reliable and light systems is becoming increasingly important in the area of avionics. SHM systems arise from the need of improving methods of evaluation and/or non-destructive testing used today [1]. The aim is to automate these processes in order to control in real time, and with as minimal human interaction as possible, all types of structures in which the proper status or operation could be a critical factor, with the increased security that this would mean. The main objectives of this study have been:

- Study of parameters which affect incorrect FBG sensor and strain gauge readings.
- Analysis of the influence of aging FBGs and strain gauges on extreme conditions of temperature and relative humidity (real conditions of use over long periods of time).
- Investigation of necessary conditioning for structural monitoring sensors in areas under extreme conditions of temperature and humidity.
- Comparison between SHM and NDE techniques for calculation of stresses and strains in aircraft structures.

FBG sensors are an optimal structural monitoring system because of the advantages offered: immunity to electromagnetic noise [2], with the consequent increase of safety and decrease of loss of information; little weight compared with traditional monitoring systems, reduced fuel consumption; wide working temperature ranges, up to 600 °C in some cases [3].

Thermography inspections on a material consist of interpreting and quantifying the temperature field of a surface. The areas with different thermal conductivities, caused by differences in the internal structure of the body or by defects, affect the circulation of an established heat flow and will cool or heat more or less rapidly than the rest [4].

## 2. Methodology

Strain gauges have been used as comparative elements due to their established used in the field of structural monitoring; moreover, a finite element analysis using ANSYS has been realized. Three F114 steel specimens have been instrumented, 1,000 × 100 × 10 mm size, with six arrays of five FBGs each, and 38 strain gauges. FBG sensors and gauges have been implemented as close together as possible, so that supported efforts are as representative as possible.

### 2.1. Strain Gauges Instrumentation

For implementation of strain gauges, parameters that could affect the readings have been taken into account, defining the nine factors shown in Table 1. These nine factors have been defined according to experienced CTA staff and strain gauge manufacturer’s recommendations. Based on the number of parameters that affect the readings of the gauges, a matrix which identifies 38 necessary gauges has been defined.

A strain gauge is a conductive wire that changes its conductivity when it is deformed, so that if we fix it to a proof we can follow the deformation of the specimen along with the gauge. Depending on the gauge K factor, which represents the variation of resistivity as a function of length, the variation of resistance in these devices is K multiplied by the variation of length:

(1) $$\frac{\Delta R}{R}=K\varepsilon$$

**Figure 1.**
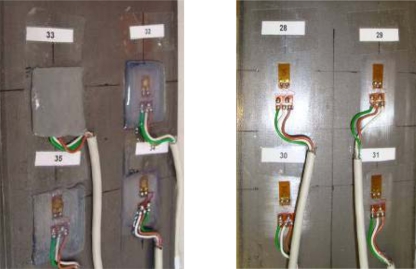
This figure shows instrumented strain gauges on both sides of Specimen 3.

A strain gauge consists of four parts: grid, support, housing and dots. It is necessary to follow these steps for implementation: surface cleaning, marking the areas of bonding, surface sanding, conditioning, preparation of the strain gauge, gluing, welding wires and cables, wiring and environmental protection [5]. Gauges 28 to 35 on specimen 3 are shown on Figure 1.

### 2.2. Fiber Bragg Grating Sensors

Fiber optic bonding requires a more elaborated process than strain gauges: the implementation surface is sanded, the area where FBG sensor is going to be placed is cleaned with isopropyl alcohol, the optical fiber and FBG sensors are fixed to the specimen areas established. Finally, the adhesive selected is elaborated, applied and let at least 24 hours. This process requires special care because of the extreme sensitivity of the optical fiber.

**Figure 2.**
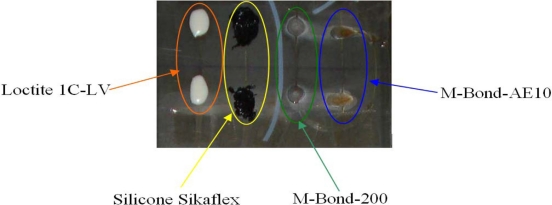
Four FBG sensors glued on Specimen 3 with four different adhesives.

**Figure 3.**
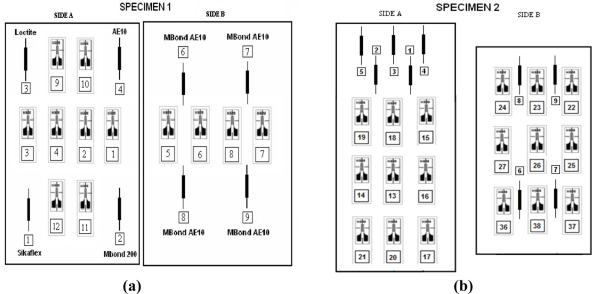

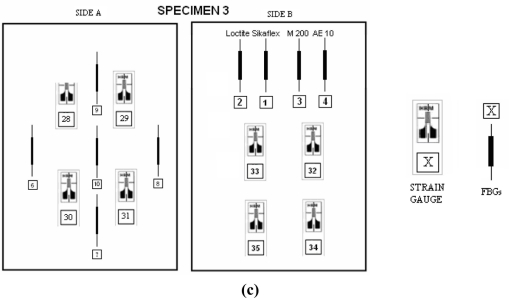
Schematic showing the lay-out instrumentation of strain gauges and FBGs on specimen 1 **(a)**, specimen 2 **(b)** and specimen 3 **(c)**.

In each specimen two optical fibers with five FBG sensors each one, one optical fiber per side, were bonded. Each specimen was treated as follows: four FBGs of one optic fiber were bonded to the specimen on one of the sides, leaving the fifth inside a Teflon tube for measurement of temperature variations [6]. On the other side of the specimens, five FBGs were applied with the most widely used adhesive, M-BOND-AE-10, made of epoxy resin and a curing agent. As seen on Figure 2, other adhesives used were SILICONE Sikaflex, M-BOND-200, LOCTITE 1C-LV and M-BOND-AE-10. The schemes of instrumentation performed is shown on Figure 3.

### 2.3. Structural Health Monitoring Tests

**Figure 4.**
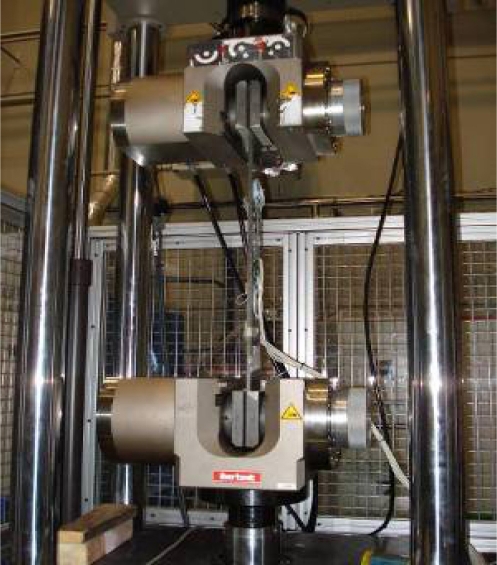
T/C machine used on tests.

The equipment used to perform the test has been a Traction/Compression machine that the CTA has in its facilities at the Technology Park of Alava (Spain). This equipment is shown on Figure 4.

This machine applies the scheduled stresses or strains through four hydraulic actuators at a maximum force of 500 kN. Load levels chosen for the tests were such that there is no risk of causing plastic deformations on specimens and test safety was not compromised.

The FBG sensors model used was OS1200 from Micron Optics Company [7]. FBG sensors reflect a wavelength that depends on the period of the grating structure. This is called Bragg wavelength (λ_B_), and it is calculated as:

(2) $$\lambda_{B}=2n\Lambda$$

where n is the refractive index of the fibre core, and Λ is the period of the grating. Changes in strain (Δε) or temperature (ΔT) produce changes in Λ, which can be noticed by measuring the corresponding changes in the wavelength of the reflected light. The equation that relates all these changes is:

(3) $$\Delta \lambda_{B}=K_{\varepsilon }\Delta_{\varepsilon }+K_{T}\Delta_{T}$$

where Kε and K_T_ are constants. In the FBG sensors used in this work, Kε was 1.25 pm/μstrain and K_T_ was 10 pm/°C [8]. As interrogator device the SM130–200 system from the Micron Optics Company was employed. It has two channels and an interrogation rate of 100 Hz. This device can be used in the range of wavelengths between 1,510 and 1,590 nm. For this study we have selected these five wavelengths for each one of the two channels: 1,526–1,536–1,546–1,556–1,566 nm. The interrogator is able to sense up to 250 different gratings in each channel, with a measurement resolution of 0.5 pm [9].

Two series of tests have been accomplished, one before the aging of the specimens and after aging them. In the first series traction, compression and fatigue tests were performed on the three samples:

- SPECIMEN 1: 200 kN Traction–5 kN Compression–3,500 fatigue cycles (20–180 kN)
- SPECIMEN 2: 200 kN Traction–5 kN Compression–18,000 fatigue cycles (20–180 kN)
- SPECIMEN 3: 200 kN Traction–5 kN Compression–73,500 fatigue cycles (20–180 kN)

**Figure 5.**
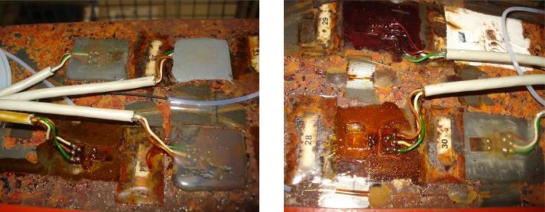
State of the gauges and FBGs after aging on climate chamber.

Static loads have been applied on steps of 20 kN until 200 kN on traction, and steps of 0.5 kN to 5 kN on compression. After the first series the specimens 2 and 3 were aged for 60 and 30 days respectively, leaving specimen 1 without aging. The state of the specimen aged is shown on Figure 5.

The second series of tests consisted of traction and compression tests on the three specimens with the loading levels discussed above. When performing the pretest with specimen 1, there was a breakage after FBGs number 6, with the consequent invisibility of FBG sensors 7, 8, 9 and 10.

### 2.4. Infrared Thermography Techniques for Tension-Field Measurement

**Figure 6.**
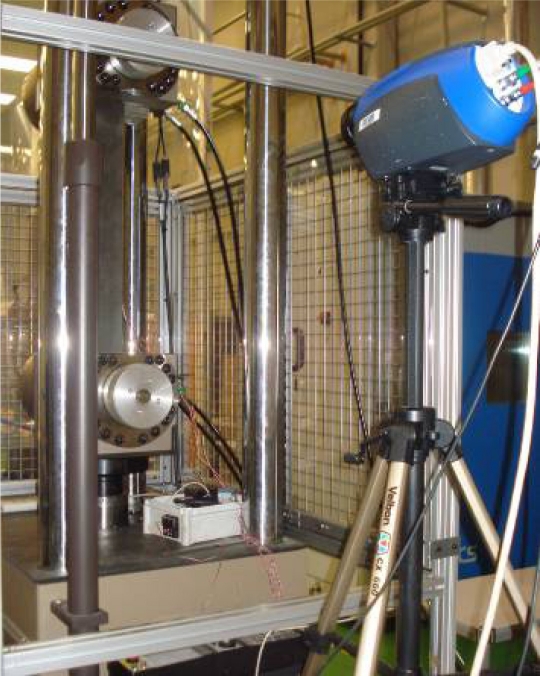
Layout of thermography tests.

In a second phase of the tests, Infrared Thermography techniques have been used to calculate forces on a fourth specimen with the same specifications than used with FBGs and strain gauges. It has been instrumented with four strain gauges on one side and cleaned on the other side, achieving inspections of IT of fatigue stresses and comparing with data obtained from gauges. The tests have been made after aging. Lay-Out of the test is shown on Figure 6. The model of the imager used in the tests is SILVER 480M with the following characteristics:

- 320 × 256 element InSb detector (pitch: 30 μm).
- Wavelength range: 3.6–5.1 μm
- Thermal sensitivity: NEDT at 25 °C < 20 mK.
- Speed: 380 Hz to full-frame and windowing options up to 20,000 Hz
- System lock-in integrated on camera.
- Stress analysis software.

Every material, depending on its temperature, emits an electromagnetic radiation whose wavelength depends on temperature. The range of wavelengths on this study is in the infrared band of the electromagnetic spectrum, so the measuring devices must be capable of detecting radiation in this band of spectrum. Another important factor when we measure stresses is the emissivity of the body, which is a characteristic of the material surface. In general, we can summarize that the higher the temperature at which a body is exposed the shorter its radiation’s wavelength is. When providing energy to a material as tension its temperature increases slightly, so knowing its emissivity makes it possible to know the range of temperatures where the specimen moves. Using data processing software, and using as inputs the emissivity of the material and the frequency of cycling, we can calculate the stresses to which the material is subjected.

The specimen has been subjected to different types of stress fatigue, varying the frequency of cycling and stress limits:

▪ Test 1: 10–210 kN
▪ Test 2: 10–310 kN
▪ Test 3: 40–340 kN (E-MODE)
▪ Test 4: 60–360 kN (E-MODE)
▪ Test 5: 40–340 kN (D-MODE)
▪ Test 6: 60–360 kN (D-MODE)

These six tests were done coordinating the frequencies of fatigue and the frequency of acquisition of IT camera. The main objective of these trials was to study the effect of emissivity of the material and the frequency in the results. Two different acquisition systems have been used to acquire data from strain gauges, TAUCON and HBM models. Both of them have been used in every test and their performance working with different frequencies of acquisition, from 5 to 10 Hz, compared. Usually the thermographic software which calculates the stress has a fast Fourier analysis module (FFA) for non-purely sinusoidal periodic loads. This feature allows the compilation of the harmonics of the load.

### 2.5. Finite Element Simulation

A FEM simulation 3D study has been performed, using ANSYS v.12 analysis software. This study has been used to make a second comparison, this time between FBG sensors and thermography. Meshing has been done with 20-node hexahedrons elements using an elastic linear calculating method.

**Figure 7.**
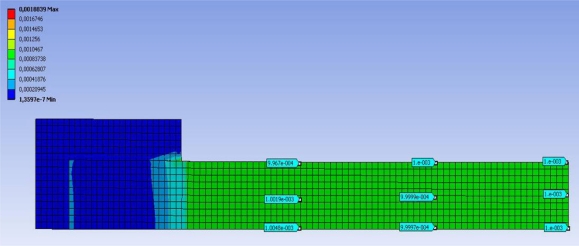
FEM strain analysis. The image shows a quarter of the specimen; results of other three quarters are identical due to the double symmetry of the specimen.

There were two studies, one studying deformations and other one calculating strains (Figure 7). The studied tensile effort has been 200 kN simulating the jaws of the T/C machine tying the specimen 100 mm from each end. The expected deformation for the induced load is 1,000 με, obtaining values between 996 με and 1,005 με.

## 3. Results and Discussion

### 3.1. FBGs and Strain Gauges

Depending on the adhesive used, the response of each FBG varies significantly. The glue which has given better results before and after the aging has been M-BOND-AE-10. The Sikaflex glue has produced misreadings of every FBG in which it was present, not being in many cases the tension increases applied and microstrain lectures obtained from FBGs proportional.

In the first test no comparator parameter has worsened the readings of strain gauges, so it cannot be deduced that any of the gauges and their adhesives vary the obtained results significantly, being these results irrelevant.

**Figure 8.**
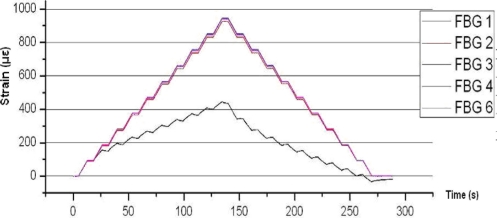
Specimen 1. Test before aging. 200 kN Traction stress. FBGs 1 to 6. Note that FBG5 is used for temperature compensation.

Figure 8 shows the results of FBGs 1 to 6 of specimen 1. As shown, FBG 2, 3, 4 and 6 are around 95% of value obtained from ANSYS FEM models. The Sikaflex glue used in FBG 1 did not give proper readings.

**Figure 9.**
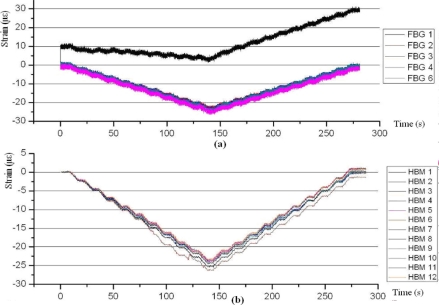
Specimen 1. Test before aging. 5kN Compression stress. FBGs **(a)** and strain gauges **(b)**.

For compression efforts (Figure 9), FBG sensors generate much more noise than strain gauges, especially if the stresses are small. The readings of Sikaflex glue can be ignored, since the zero of the beginning of the test is 10 με and proportionality of microstrains is altered if the load is increasing or decreasing. With respect to the gauges the results obtained are very similar with both the TAUCON and HBM acquisition system. However, the noise generated by HBM is much lower than TAUCON. Strain gauge number 2 obtained worse readings than the other gauges; the reason of this misreading is the model of the gauge, CEA-06-125UW-350.

**Figure 10.**
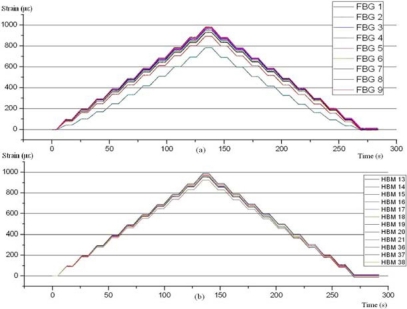
Specimen 2. Test before aging. 200 kN Traction stress. FBG **(a)** and strain gauges **(b)**.

The results of the first tests on specimen 2 can be seen in Figure 10. All readings are as expected, except the FBG 4 and the strain gauge 17. These two sensors are located one above the other and the difference in readings with the other sensors is less than 15%. This may be due to a slight deviation in the alignment in the jaws of the specimen in the T/C machine, since the FBG 5 and gauges 14, 19 and 21, located on the opposite side, give readings slightly above the rest.

**Figure 11.**
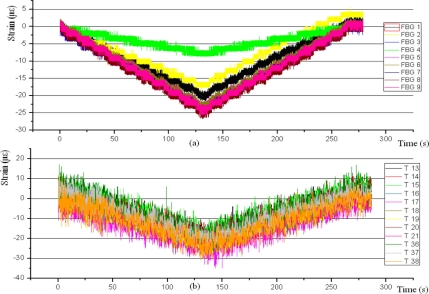
Specimen 2. Test before aging. 5 kN Compression stress. FBGs **(a)** and strain gauges **(b)**.

Figure 11 shows the FBG of specimen 2, subjected to compression, providing erroneous results. The worst case is given by FBG 4, being also wrong the FBG 1 and 2 results. As for gauges, observed noise is rather high; this is due to the Taucon acquisition system. HBM tests gave the same results but with less noise. Note that gauge T15 does not start on zero microstrain, this lecture thus being invalid. The error is due to the model of terminals used, CEG-60D IY.

**Figure 12.**
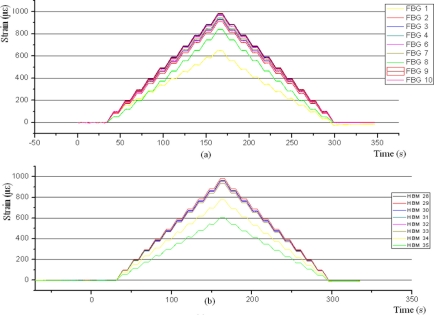
Specimen 3. Test before aging. 200kN Traction stress. FBG **(a)** and strain gauges **(b)**.

In the tensile tests on specimen 3 (Figure 12), for the four FBG sensors glued on side 1 all the adhesives provide accurate readings, except Sikaflex, on FBG 1. FBG 8 has been glued with M-BOND-AE-10 and the reading was significantly lower than the rest. This error may be due to an incorrect implementation, since the sensor was on the edge of the specimen and its positioning was very difficult. The readings of the gauges 34 and 35 are below than the rest. The item they have in common is the protection. This protection is from the same manufacturer in both cases, although different models, RTV-3140 and RTV-3145.

**Figure 13.**
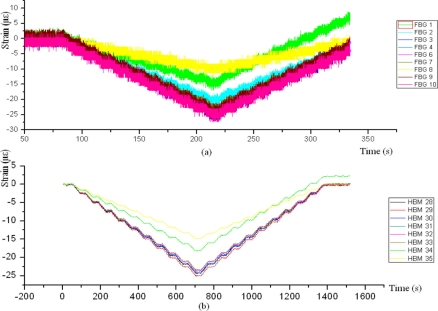
Specimen 3. Test before aging. 5kN Compression stress. FBGs **(a)** and strain gauges **(b)**.

Figure 13 shows the results of tests of the specimen 3 under compression load. As in traction, protection of gauges 34 and 35 is ineffective for this type of test. In FBGs 1 and 8, there are higher readings than the rest. As mentioned previously these high readings are due to the Sikaflex adhesive in FBG 1 and in FBG 8, to a difficult implementation.

**Figure 14.**
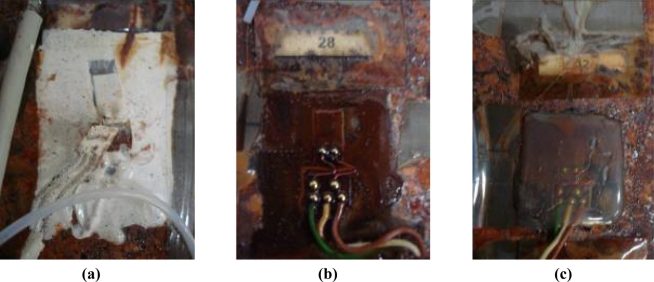
State of gauges after aging. The strain gauge 31 can be seen almost detached but acquiring **(a)**; strain gauges 28 **(b)** and 32 **(c)** are not affected by aging.

After aging the specimens 2 and 3 for 30 and 60 days, respectively, at 70 °C and 90% RH, breaks and cuts appeared in both the fiber-optic networks and strain gauges. These breaks are motivated by the emergence of many corrosion points generated in the specimens, as well as the high temperature and humidity, which are an important factor in the conservation of the gauges. The state of some aged gauges is shown on Figure 14.

During handling FBG 3 in specimen 1 was damaged, producing a different reading in test 2. As expected the FBGs 2, 4 and 5 have not undergone any change and the Sikaflex FBG 1, has again given poor results. As for the gauges, it was impossible to obtain any result from gauge 11 and results from gauge 12 have been negative.

**Figure 15.**
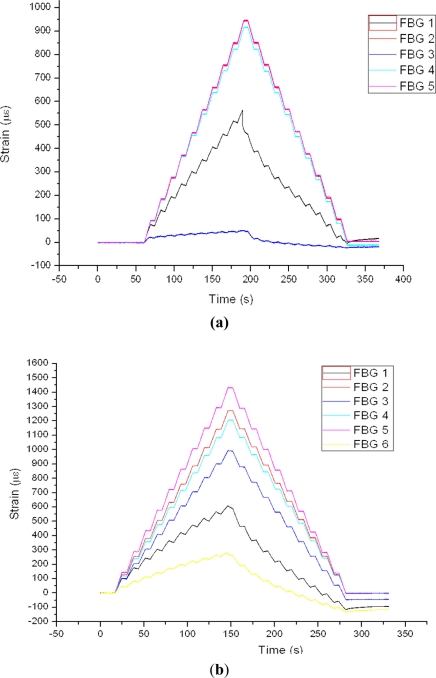

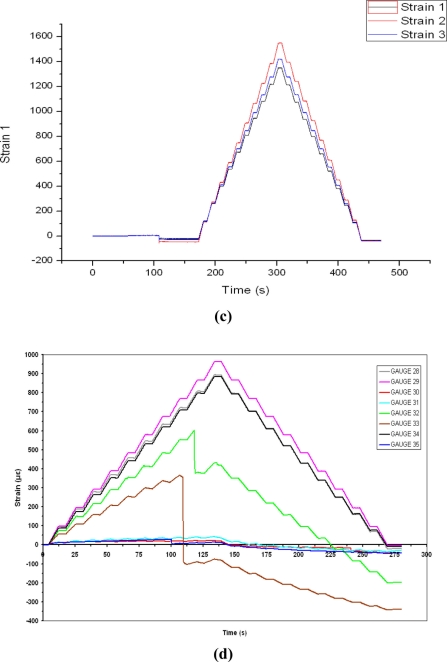
Test after aging. 200 kN Traction stress. This figures display FBGs on specimen 1 **(a)**, FBGs on specimen 2 **(b)**, FBGs on specimen 3 **(c)** and strain gauges on specimen 3 **(d)**.

The Figure 15 shows the results for FBG sensors of the three specimens with traction stress and after the aging of specimens 2 and 3. The results of the tests on aged specimens 2 and 3 were significant. The FBG sensor readings have been considerably higher than those obtained before aging, and the 1,400 microstrains obtained are far from the 950 microstrains expected.

The effect of high temperature has been ruled out because the FBG model tested is capable of withstanding temperatures up to 120 °C. Therefore, the factor that caused the negative effect on the FBG is the high humidity.

There are some studies about the effect of relative humidity on the behavior of FBG sensors in the short-term [10]. In short term, for a maximum of 12 hours, the effects are considerably less than long term, and when the exposition is finished those effects disappear.

With regard to the protections of strain gauges, it is noteworthy that the best results have been with M-COAT-B protection, the result being exactly the same before and after aging. The results of the protections M-COAT-A and PU 140 have been slightly lower after staying in the climate chamber. Protections M-COAT-C, M-COAT SG250-D and SG250 have been proven unsuitable for long exposures to extreme conditions. In the case of strain gauges 32 and 33, their protection did not properly protect gauges. In fact, strain gauges 32 and 33 stopped working properly during the test.

### 3.2. Thermography

The readings of tensile-fields obtained using IT techniques inspections are not as accurate as FBG’s. It was noted that the emissivity of the material has a major effect when calculating stresses. Average errors are about 15% for emissivities between 0.7 and 1. The load applied to the specimen and the read by the strain gauges has been 210 kN, however the calculated load for an emissivity of 0.7 has not reached 180 kN and the corresponding emissivity 1 has exceeded 250 kN.

**Figure 16.**
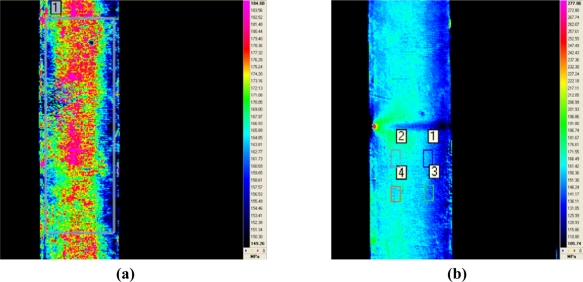
Thermograms acquired during the IT tests. In (b) can be seen a slot created in the opposite side for detect inspection.

**Figure 17.**
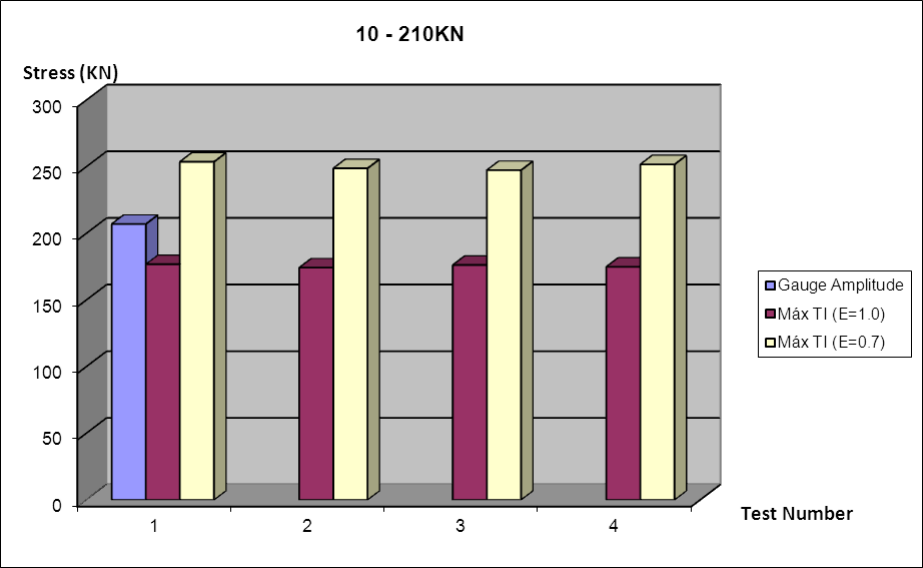
Stress comparison between strain gauges and IT techniques.

The position of the four gauges used is shown on the second thermogram of Figure 16. The comparison between gauges and IT techniques is shown on Figure 17.

Fatigue tests were performed with sinusoidal loads frequency in a spectrum analysis of 2 Hz and none has shown any change in the calculation of stresses, so it is expected that it occurs at higher frequencies. Another important factor in the calculation of stresses using this technique is the ambient temperature, as small variations of temperature influence the results in a determinant way.

## 4. Conclusions

The behavior of FBGs on monitoring systems for long periods of time could be a problem if they are not correctly protected and if the adhesives are not properly selected. The main drawback found is the relative humidity, a factor which, if not appropriately isolated, can make our system provide erroneous data. Due to this agent, readings variations up to 65% higher than expected were observed. It is therefore necessary to correctly insulate all the fiber in both high humidity environments and monitoring systems working for long periods of time. As a solution there are several methods like isolating the optical fiber in its entirety and gluing FBG sensors completely, not only at the ends. A long stay at high relative humidity can affect the polyamide coating on glass fibers, resulting in contact with glass. Once in contact with glass, and after a long period of exposure, humidity can penetrate until getting in contact with Bragg gratings, making their refractive index vary. An increase in “n” produces a shift in the peak frequency of the Bragg Grating and giving readings much higher than the real values.

In the case of the strain gauges, the protection which best worked was M-COAT-B, with M-COAT-A and PU 140 being almost as good. In the calculation of stresses in fatigue with IT techniques an average error of 15% is observed, being the emissivity of the material a very influential factor.

## Figures and Tables

**Table 1.** Different parameters that may affect the reading of gauges.

**Gauge Type**	**Adhesive**	**Terminals**	**Thread**	**Thread Length**	**Wire**	**Wire Length**	**Wiring**	**Protection**

CEA-13-125UW-350	M-BOND 200	CPF-75D IY	134-AWP	134AWP	DATAX 3 × 0.22 AP.	DATAX 3×0.22 AP.	3 CABLES	M-COAT A

CEA-06-125UW-350	M-BOND AE-10	CPF-75C II	134-AWN-W	134AWP 10cm	DATAX 3 × 0.25	DATAX 3×0.22 AP. 50 m	2 CABLES	M-COAT B

1-LY41-1.5/350	M-BOND 600/610	CEG-60D IY	EPDX		DATAX 25 × 0.22		4 CABLES	M-COAT C
		
1-LY41-3/350 1-LY13-3/350	Z-70 X 280	1-LS 5 WITHOUT TERMINALS			326-BSV 330-FFE			M-COAT D RTV-3140
		
1-LY47-3/350					Kab.4.1/00.3			RTV-3145
1-LY48-3/350 1-LY61-3/120								PU 140 SG250
